# Author Correction: Selection and horizontal gene transfer underlie microdiversity-level heterogeneity in resistance gene fate during wastewater treatment

**DOI:** 10.1038/s41467-024-50577-6

**Published:** 2024-07-22

**Authors:** Connor L. Brown, Ayella Maile-Moskowitz, Allison J. Lopatkin, Kang Xia, Latania K. Logan, Benjamin C. Davis, Liqing Zhang, Peter J. Vikesland, Amy Pruden

**Affiliations:** 1 Dept. of Civil and Environmental Engineering, Virginia Tech, Blacksburg, USA; 2 Dept. of Chemical Engineering, University of Rochester, Rochester, USA; 3 School of Plant and Environmental Sciences, Virginia Tech, Blacksburg, USA; 4 Dept. of Pediatrics, Emory University, Atlanta, USA; 5 Office of Research and Development, U.S. Environmental Protection Agency, Cincinnati, USA; 6 Dept. of Computer Science, Virginia Tech, Blacksburg, USA

**Keywords:** Water microbiology, Phage biology, Metagenomics

Correction to: *Nature Communications* 10.1038/s41467-024-49742-8, published online 26 June 2024

In this article the affiliation details for Benjamin C. Davis were incorrectly given as ‘Dept. of Pediatrics, Emory University, Atlanta, USA’ but should have been ‘Office of Research and Development, U.S. Environmental Protection Agency, Cincinnati, USA’. Also, the affiliation details for Latania K. Logan were incorrectly given as ‘Office of Research and Development, U.S. Environmental Protection Agency, Washington, USA’ but should have been ‘Dept. of Pediatrics, Emory University, Atlanta, USA’. The original article has been corrected.

