# Supplementary material for: Selection and horizontal gene transfer underlie microdiversity-level heterogeneity in resistance gene fate during wastewater treatment
Source: Nat Commun. 2024 Jun 26;15:5412. doi: 10.1038/s41467-024-49742-8 (PMC11208604; doi:10.1038/s41467-024-49742-8)
Supplement: Supplementary file 1 — Supplementary Information [file 41467_2024_49742_MOESM1_ESM.pdf]

Selection and horizontal gene transfer underly microdiversity-level heterogeneity in the fate of resistance genes during wastewater treatment

Connor L. Brown[1], Ayella Maile-Moskowitz[2], Allison J. Lopatkin[3], Kang Xia[4], Latania Logan[5], Benjamin C. Davis[2], Liqing Zhang[6], Peter Vikesland[2], Amy Pruden[2]

Dept. of Genetics, Bioinformatics, and Computational Biology, Virginia Tech[1]
Dept. of Civil and Environmental Engineering, Virginia Tech[2]
Dept. of Chemical Engineering, University of Rochester[3]
Office of Research and Development, U.S. Environmental Protection Agency[4]
Dept. of Plant and Environmental Sciences, Virginia Tech[5]
Dept. of Pediatrics, Emory University[6]
Dept. of Computer Science, Virginia Tech[7]

Supplementary Information

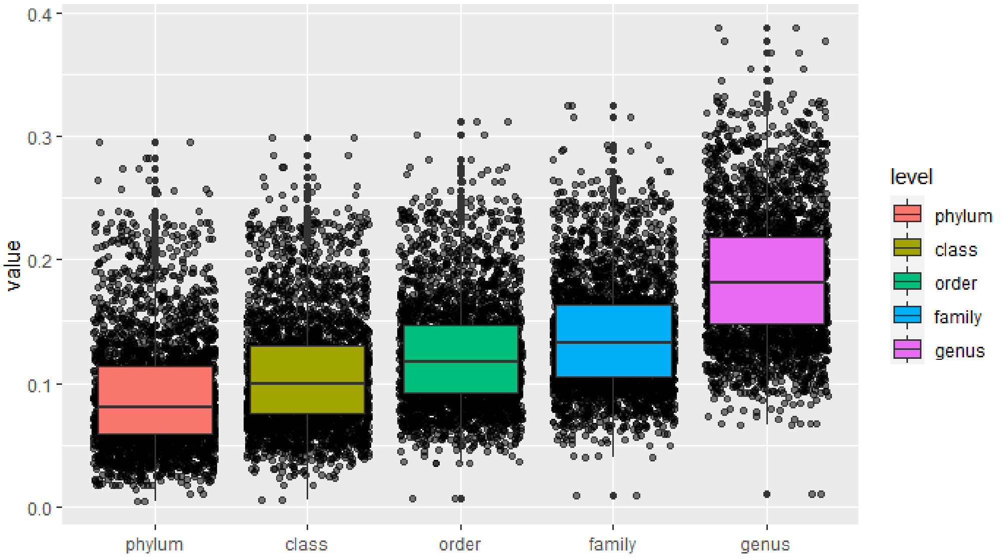

Fig S1. Pairwise Bray-Curtis distances between influent and activated sludge (AS) samples using abundances of taxa at different levels of classification. Distances between AS samples only. (B) Distances between influent samples with comparisons in the form of 10% vs. 0%.

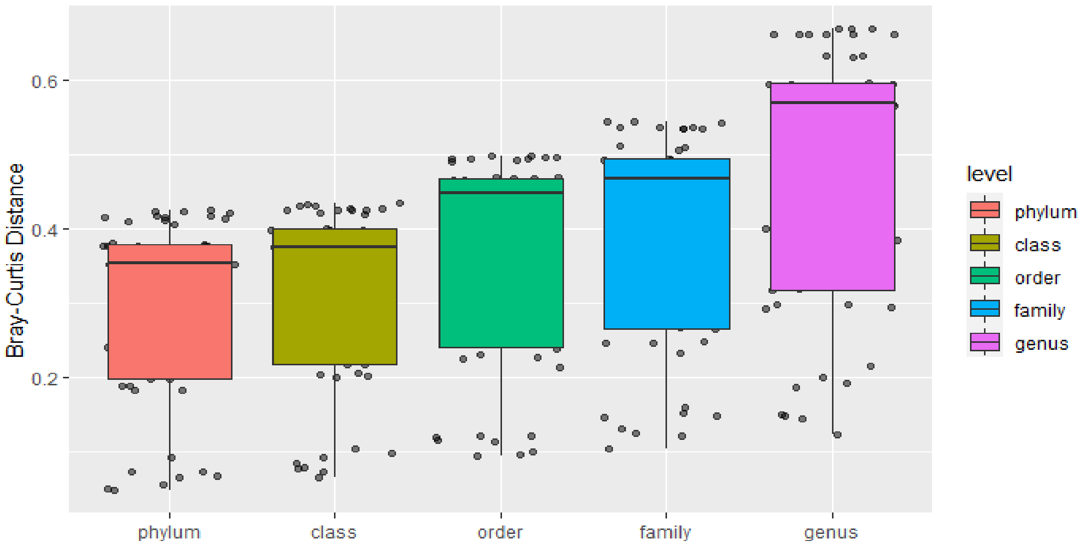

Fig S2. Pairwise Bray-Curtis distances between influent and AS samples using abundances of taxa at different levels of classification. Distances between influent samples with comparisons in the form of 10% vs. 0%.

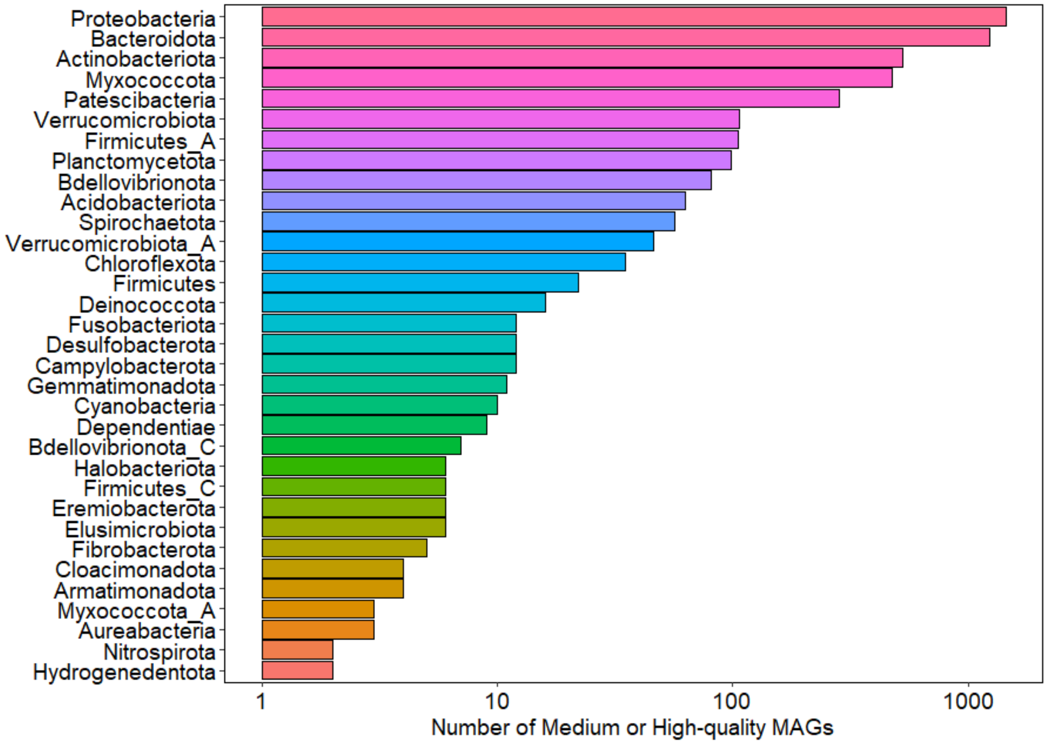

Fig. S3. Phylum-level counts of high- and medium-quality metagenome assembled genomes (MAGs) before dereplication.

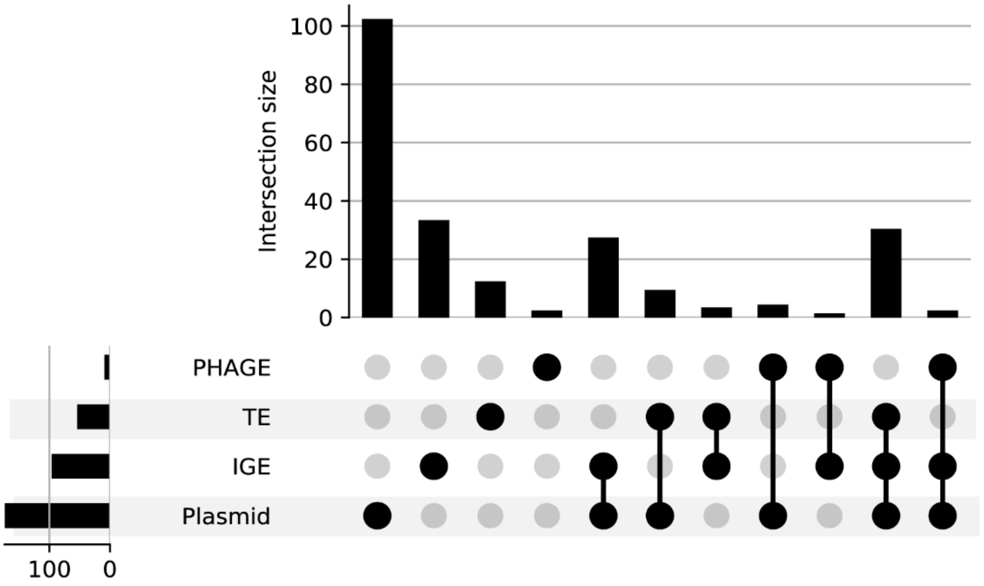

Fig. S4. Association of antibiotic resistance genes (ARGs) with putative composite mobile genetic elements (MGEs). Counts of resistance genes in terms of their co-occurring mobileOGs with different element classifications. For example, the most frequent association predicted through co-occurrence analysis was with plasmid hallmarks.

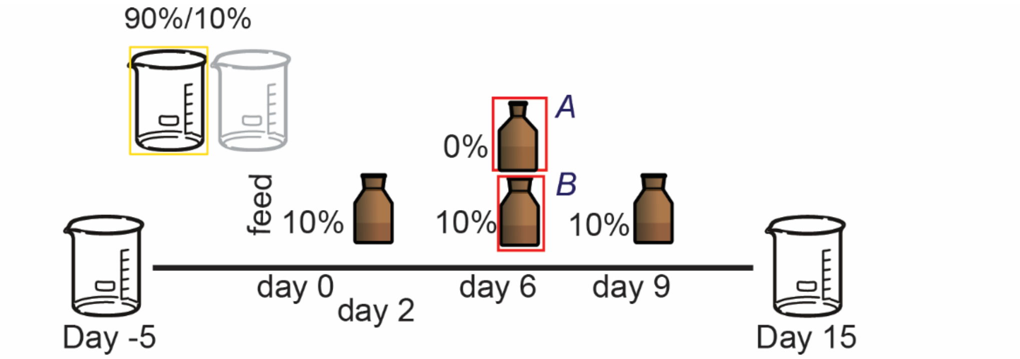

Fig. S5. Sampling scheme used for Fig. 2. Samples of feed or influent (brown bottles) and AS from reactor 10%-1 (yellow box) were deeply sequenced for use *in situ* HGT and microdiversity analyses. The 10%-specific contigs are those detected in A (day 6 0% influent/feed) but not B (day 6 10% influent/feed).

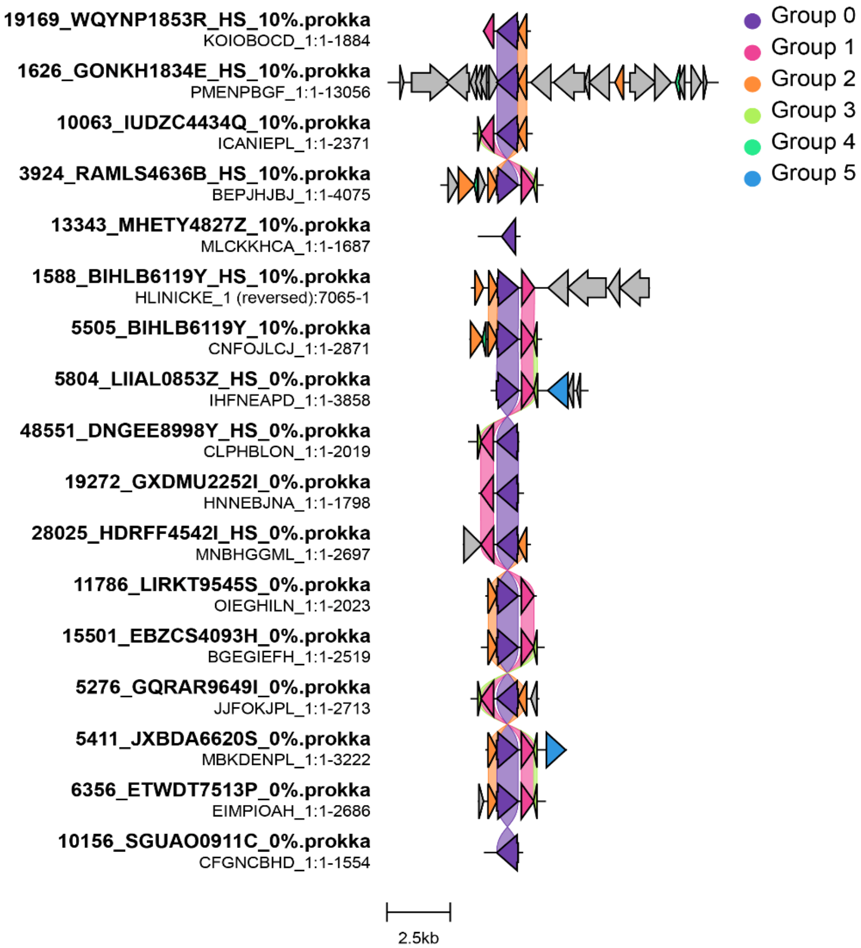

Fig. S6. Example contexts of *sul1* in the 10% or 0% influent samples. Clinker plots generated from a balanced, random sample of contigs bearing *sul1* predicted to be either native- or hospital-sewage specific.

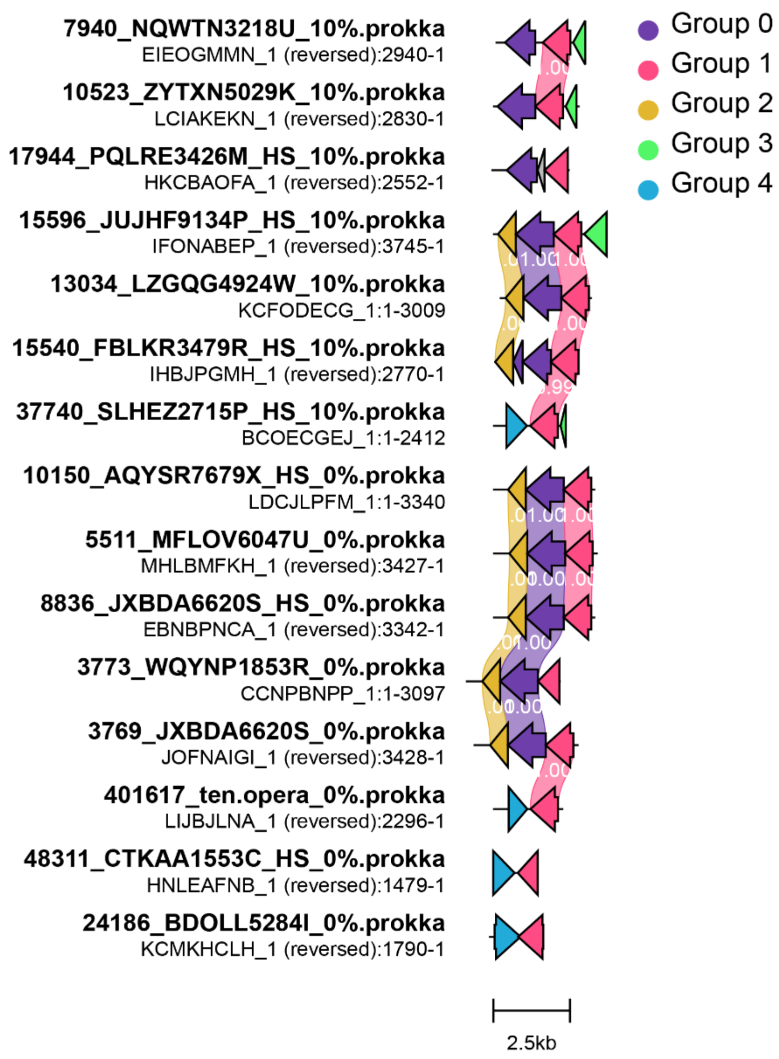

Fig. S7. Example contexts of genes encoding macrolide phosphotransferase A (*mphA*) in the 10% or 0% influent samples. Clinker plots generated from a balanced, random sample of contigs bearing *mphA* predicted to be either native- or hospital-sewage specific.

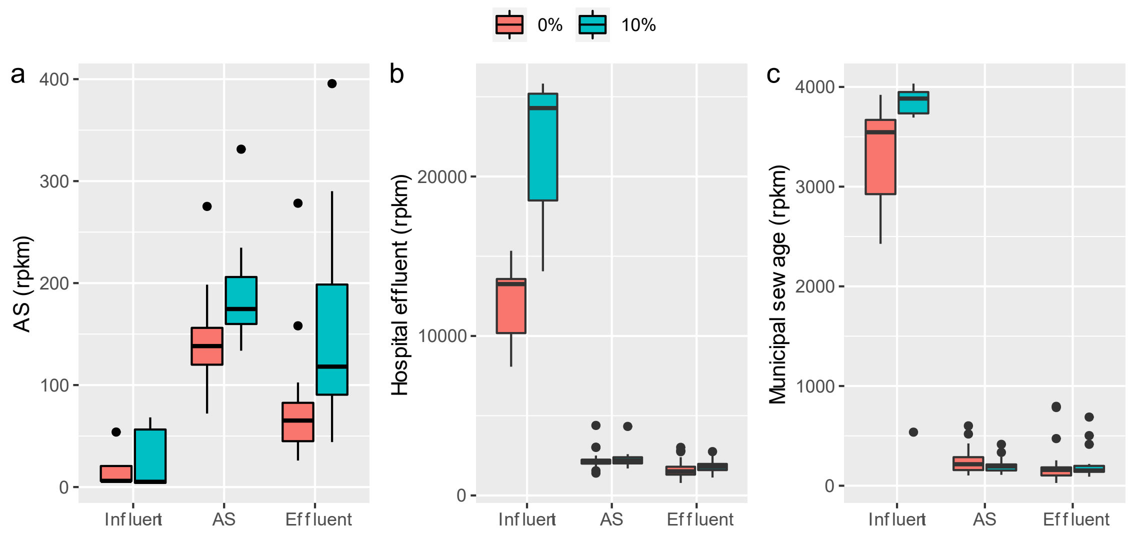

Fig S8. Relative abundance of contigs through read mapping is concordant with the Kairos assess workflow predictions. (A) Summed relative abundances of contigs predicted by Kairos assess (as in Fig. 3) to be innate to the AS microbiome. (B) Summed relative abundances of contigs predicted to be innate to hospital sewage. (C) Summed relative abundances of contigs predicted to be associated with municipal sewage.

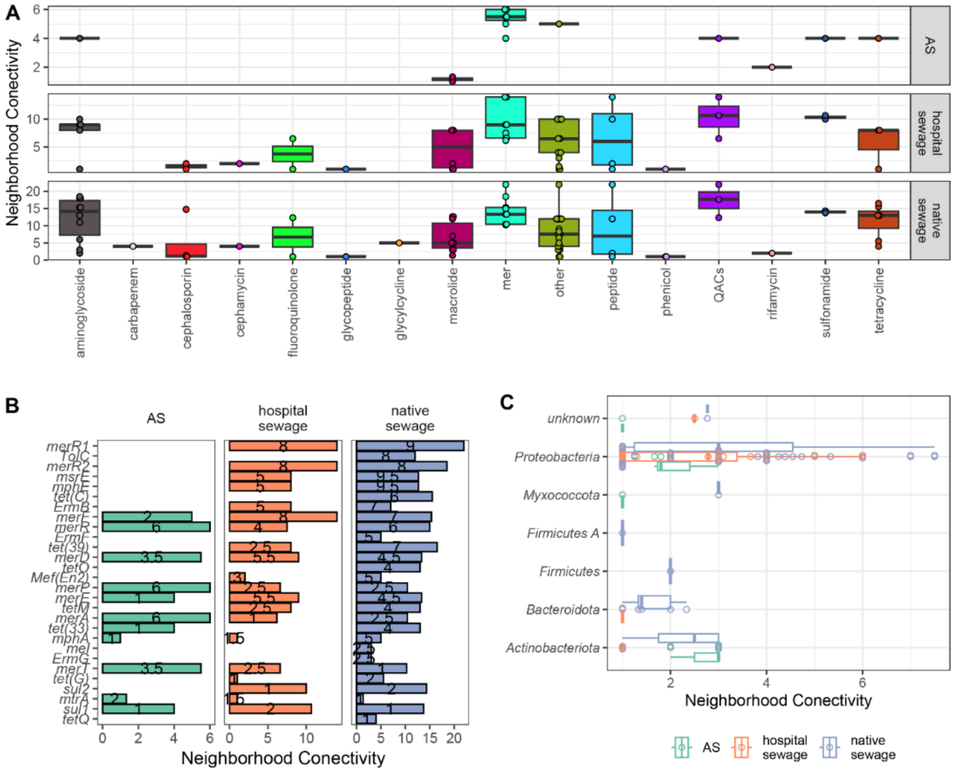

Fig. S9. Overall structure and properties of hospital- and background municipal-sewage mobile resistance gene networks were similar. (A) Drug classes showed similar rankings in terms of

neighborhood connectivity. (B) Individual resistance genes showed similar rankings in hospital sewage vs. native sewage. (C) Hospital sewage lacked many of the phyla associated with background municipal sewage.

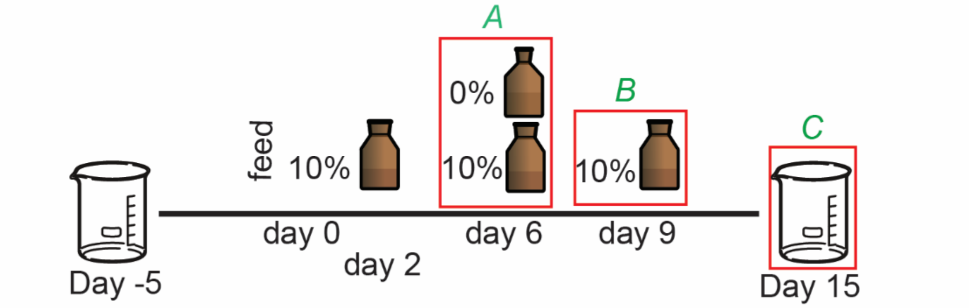

Fig. S10. Experimental and logic flow diagram for distinguishing fate of hospital and native-sewage resistance genes as in Fig. 4G. "Attenuated" genes are those present in <u>A</u> (day 6 influent/feed) but not <u>B</u> (day 9 influent/feed) or <u>C</u> (AS on the last day of sampling). "Persistent" genes are those present in <u>A</u> and <u>C</u> but not <u>B</u>.

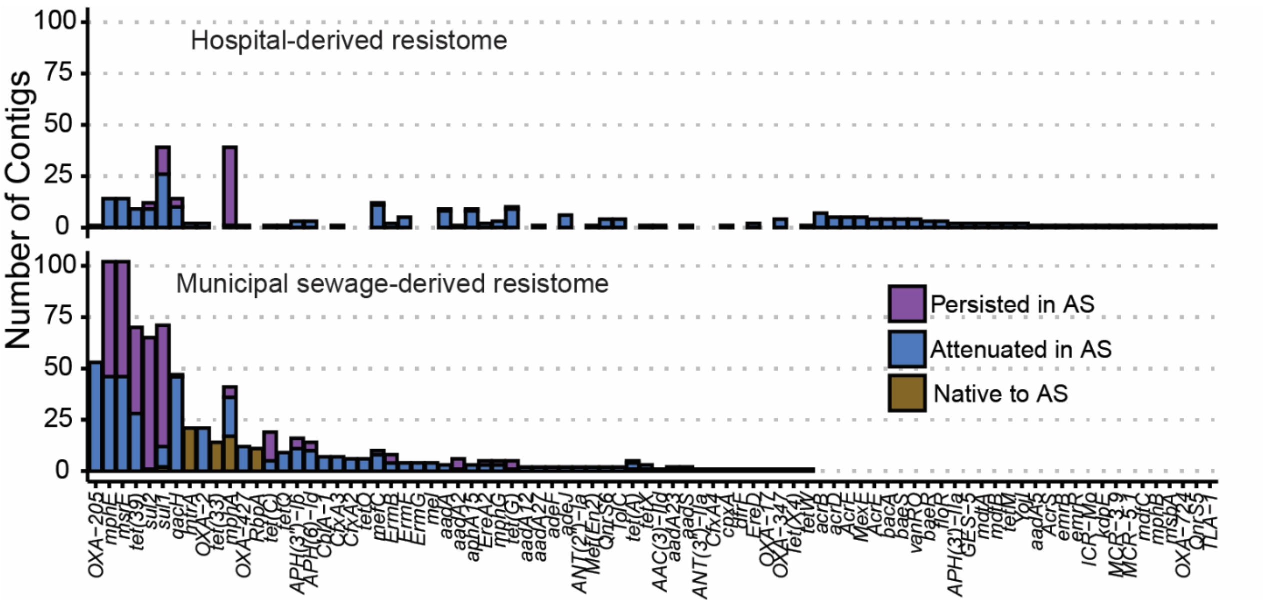

Fig. S11. Microdiversity-level disparity in the fate of hospital and native-sewage associated resistance genes. Determined using the Kairos assess workflow and logic described in Fig. S11. Persistent contigs were considered those still detectable after several days of operation. Attenuated contigs were those that were not detected in AS following several days of operation. Native contigs are described as those only detected in AS.

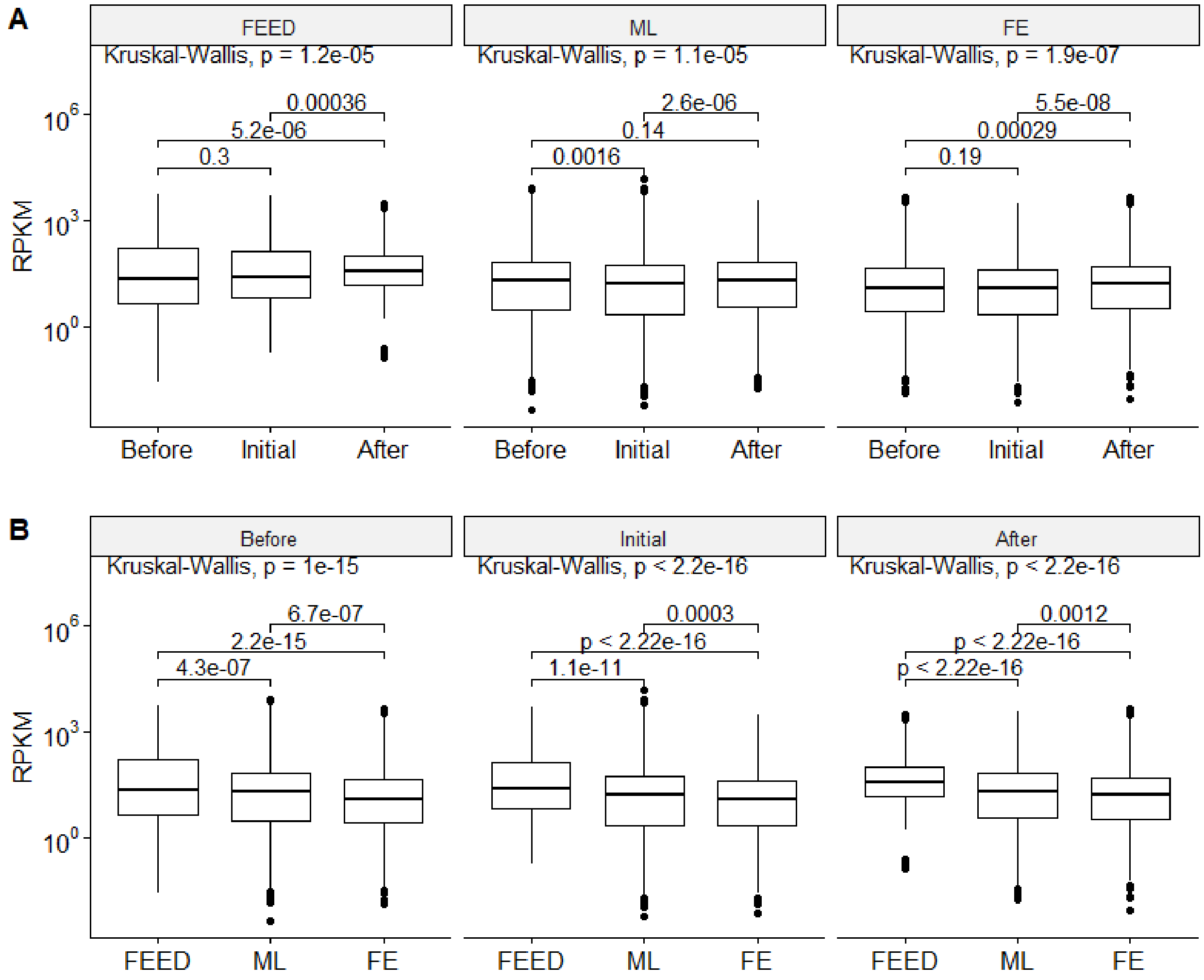

Fig. S12. Hosts of the genomes associated with attenuated resistance genes. (A) is partitioned by fraction. (B) is partitioned by date category associated with sampling. ML: mixed liquor or activated sludge. FE: final effluent. FEED: feed or influent sewage.

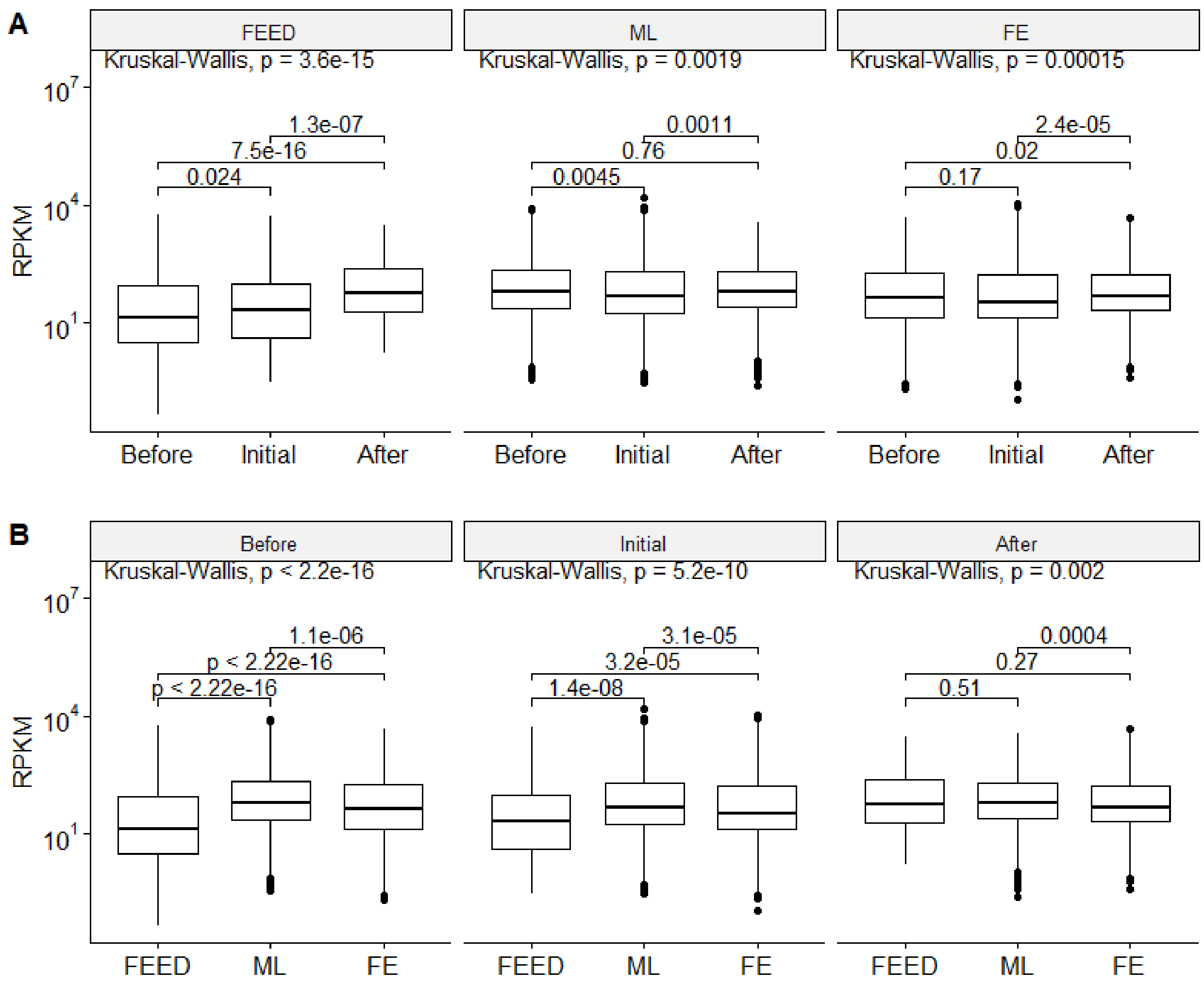

Fig. S13. Hosts of the genomes associated with persistent resistance genes. (A) is partitioned by fraction. (B) is partitioned by date category associated with sampling. ML: mixed liquor or activated sludge. FE: final effluent. FEED: feed or influent sewage.

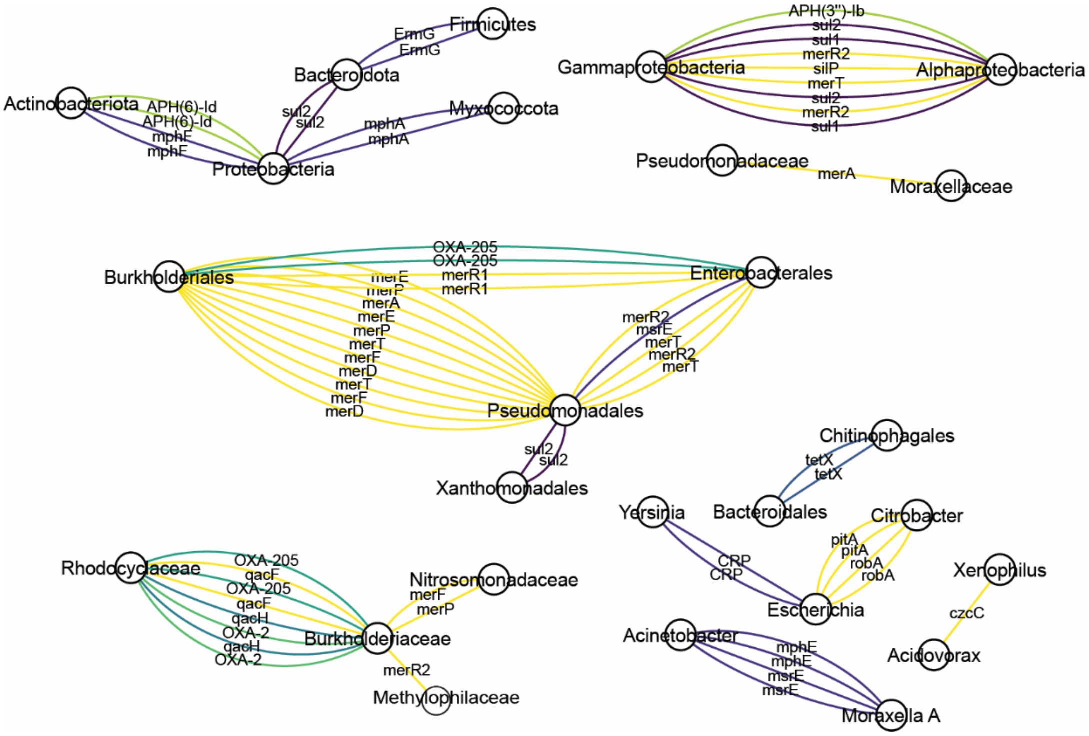

Fig. S14. Putative gene-sharing or HGT network of resistance genes. Nodes are taxa and edges are identical proteins found in contigs found predicted to be derived from those taxa.

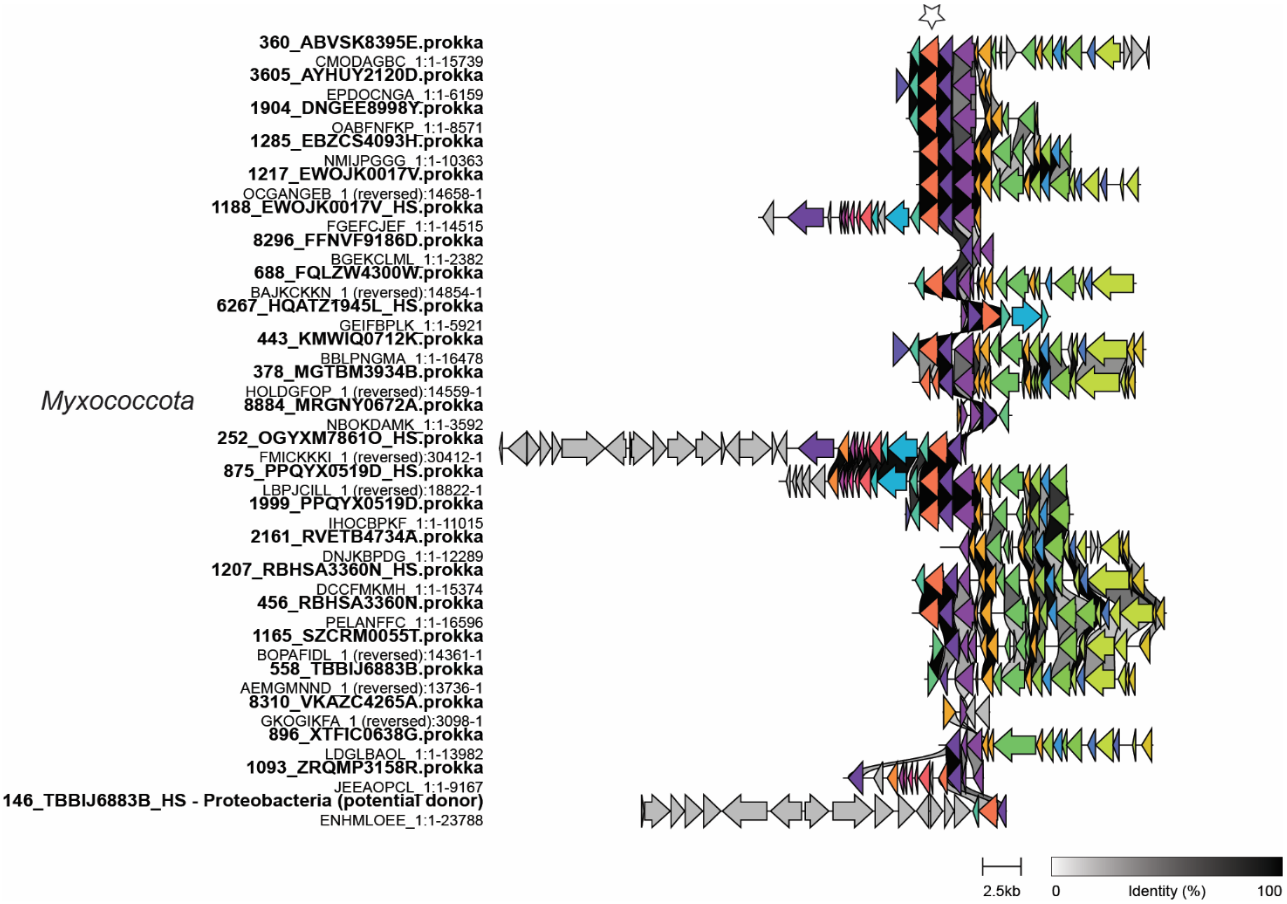

Fig. S15. Contigs associated with the putative *in situ* transfer of *mphA.* Star indicates position of *mphA*.

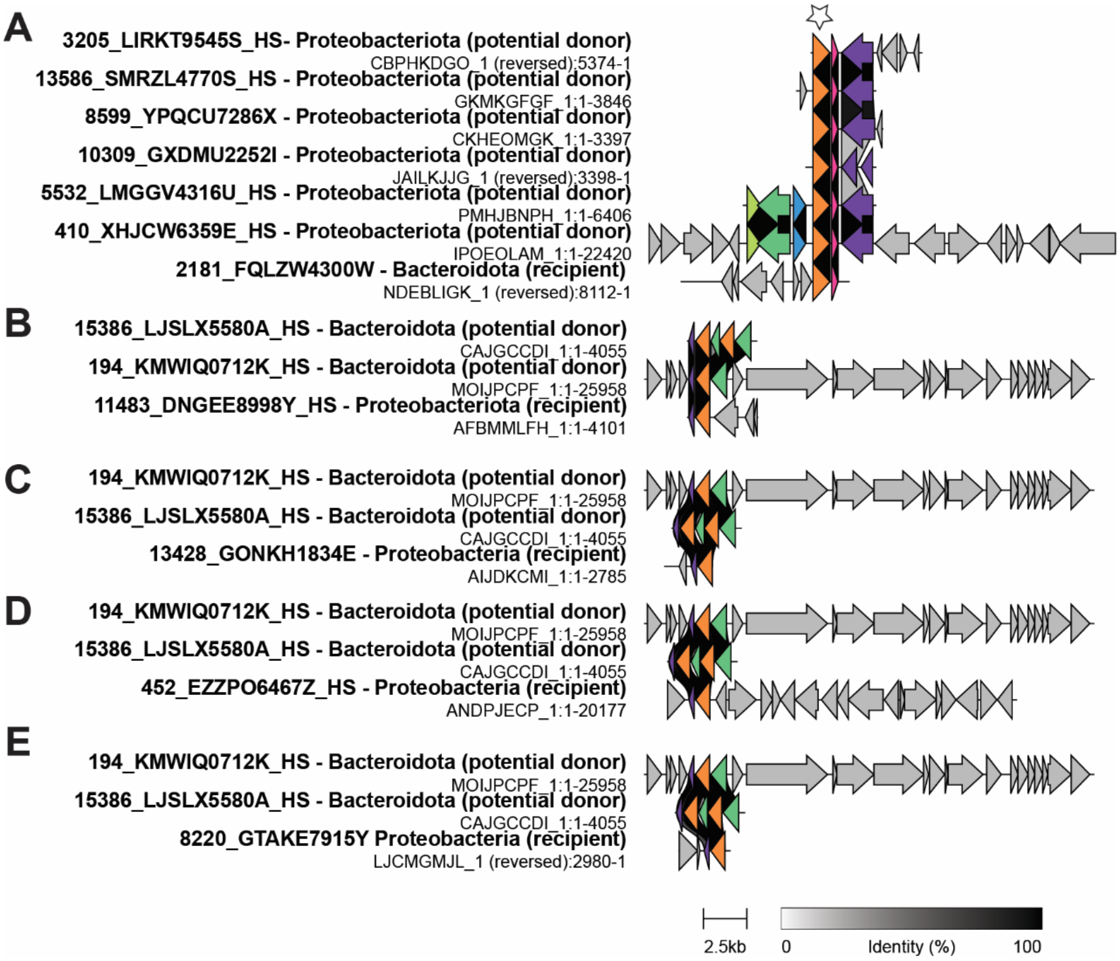

Fig. S16. Contigs were detected that predicted multiple potential pathways of *in situ* transfers of *sul2*. Start indicates position of *sul2*.

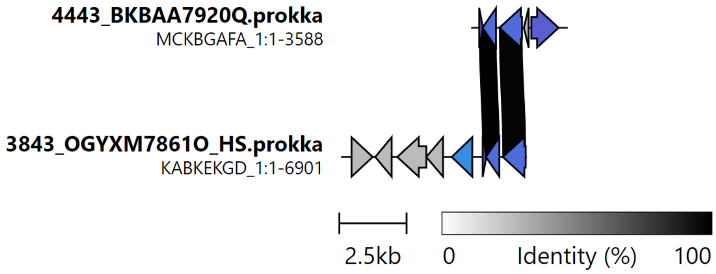

Fig. S17. Putative *in situ* HGT of *sul1* contigs. Contigs align against edges meaning that the taxonomic assignment underlying its status as an HGT may be inaccurate and/or uncertain.

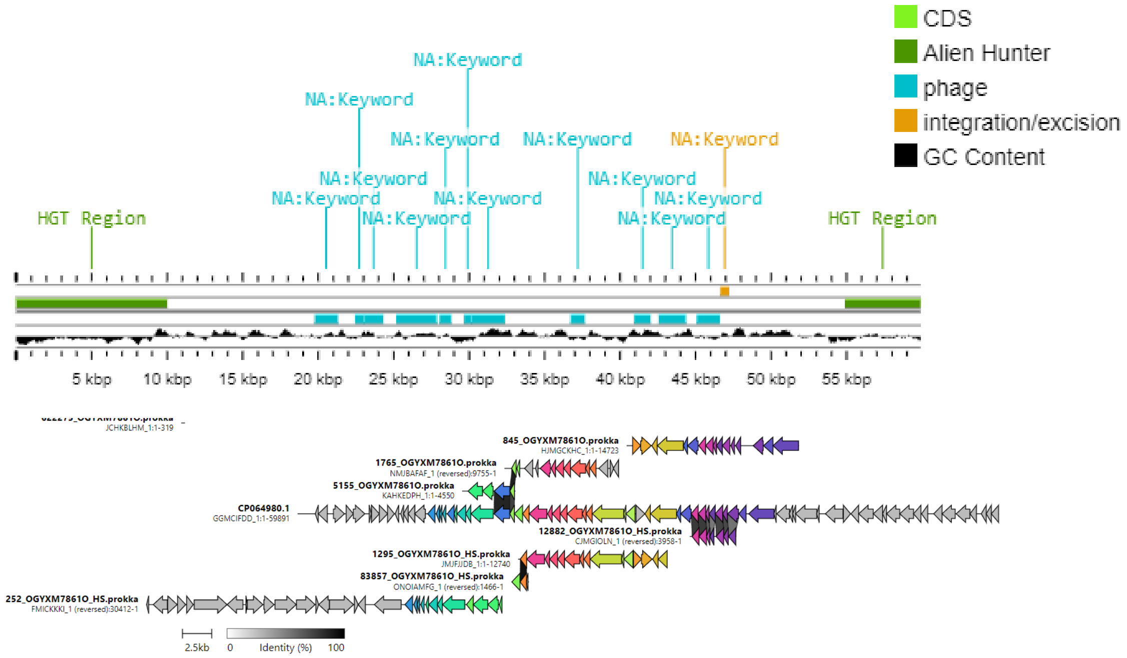

Fig. S18. Scaffolding draft phage genomes based on alignment to reference sequenced. (TOP): First 60,000 basepairs of NCBI entry CP064980.1 visualized using proksee with mobileOG-db (cyan and orange), Alien Hunter (forest green) and GC%. Alien Hunter was used to infer the prophage region as 10,000 – 55,000 bp. In this case, Alien Hunter is suggesting the region surrounding the integration site rather than the prophage is the HGT region as the prophage inhabits the majority of the 60,000 basepair fragment. (BOTTOM): Example clinker plot used to orient fragments for merging.

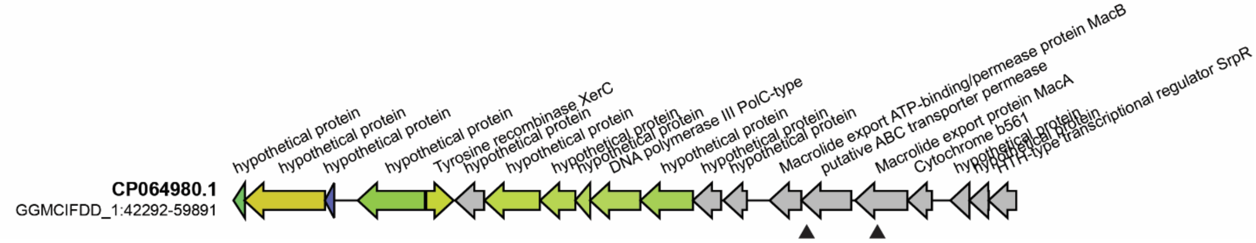

Fig. S19. One putative prophage in NCBI genome CP064980.1 was integrated near genes encoding MacA and MacB putatively associated with macrolide export.

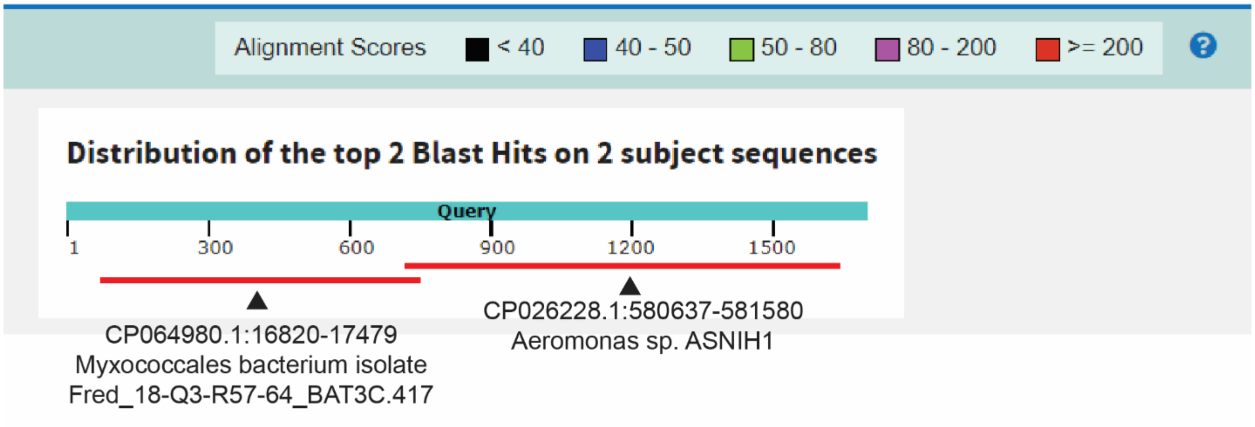

Fig. S20. Example nanopore read alignment to both *Myxococcota* and *Enterobacterales* genomes. The nanopore read here (`6de60e4c-c959-472c-93f4-1fe535ef4dd0`) encodes *mphA* as well as phage genes. Alignment details are provided in Extended Data 1.

Table S1. Hits to myxophage derived from CP064980.1.

Table S1. Hits to myxophage derived from CP064980.1.

Description	Scientific Name	Max Score	Total Score	Query Cover	E value	Per. ident	Acc. Len	Accession
Myxococcales bacterium isolate Fred_18-Q3-R57-64_BAT3C.417 chromosome	*Myxococcales bacterium*	1.07E+05	1.07E+05	100%	0	100	4018971	CP064980.1
Corallococcus sp. EGB chromosome, complete genome	*Corallococcus sp. EGB*	5012	38100	68%	0	86.62	9431171	CP079946.1
Archangium violaceum strain SDU34 chromosome, complete genome	*Archangium violaceum*	2870	17434	41%	0	77.94	12677854	CP069338.1
Myxococcus xanthus strain GH3.5.6c2 chromosome, complete genome	*Myxococcus xanthus*	2686	10077	34%	0	74.25	9321034	CP017169.1
Myxococcus xanthus strain DK101 chromosome	*Myxococcus xanthus*	2676	36430	51%	0	74.3	9346413	CP104803.1
Myxococcus xanthus DZ2 chromosome, complete genome	*Myxococcus xanthus DZ2*	2676	36430	51%	0	74.3	9365783	CP080538.1
Myxococcus xanthus strain ATCC 27925 chromosome, complete genome	*Myxococcus xanthus*	2676	36425	51%	0	74.3	9346321	CP080534.1
Myxococcus xanthus DZ2 chromosome, complete genome	*Myxococcus xanthus DZ2*	2676	36406	51%	0	74.3	9359382	CP070500.1
Myxococcus xanthus strain DK1622:pDPO-Mxn116-Pvan-Tpase chromosome, complete genome	*Myxococcus xanthus*	2676	12994	44%	0	74.3	9201039	CP065375.1
Myxococcus xanthus DK 1622, complete genome	*Myxococcus xanthus DK 1622*	2676	12994	44%	0	74.3	9139763	CP000113.1
Myxococcus xanthus strain DK1050 chromosome	*Myxococcus xanthus*	2673	23741	50%	0	74.35	9253193	CP104804.1
Myxococcus fulvus 124B02, complete genome	*Myxococcus fulvus 124B02*	2671	12002	43%	0	74.3	11048835	CP006003.1
Myxococcus sp. MH1 DNA, complete genome	*Myxococcus sp. MH1*	2625	13929	48%	0	74.19	10778154	AP026947.1
Corallococcus macrosporus DSM 14697 chromosome, complete genome	*Corallococcus macrosporus DSM 14697*	2580	24668	50%	0	74.05	8973512	CP022203.1
Myxococcus xanthus strain R31 chromosome, complete genome	*Myxococcus xanthus*	2074	12564	41%	0	75.41	9251369	CP068048.1
Stigmatella aurantiaca DW4/3-1, complete genome	*Stigmatella aurantiaca DW4/3-1*	1855	18108	36%	0	75.88	10260756	CP002271.1

Supplementary Methods

Section S1. Scaffolding phage genomes

Draft phage genomes were constructed by aligning scaffolds produced by hybrid assembly to a 45,000 basepair putative prophage extracted from CP064980.1 (pos 10,000-55,000 bp). Contigs with alignments

to the reference genome were visualized and the appropriate orientation and order inferred from the clinker[1] plot. Sequences were merged and/or reversed as appropriate using seqkit.[2]

Section S2. Suspect Screening for PPCPs

Extraction and cleanup of water samples using solid phase extraction (SPE)

An aliquot of 200 mL water sample pre-filtered with a 0.7 µm 55 mm in diameter glass fiber filter (Whatman, Maidstone, UK) was loaded onto a solid phase extraction (SPE) hydrophilic-lipophilic balance cartridge (Oasis HLB cartridges (60 mg, 3cc), Waters, Milford, MA) that was pre-conditioned with 3 mL HPLC-grade methanol and 3 mL ultra-pure water. The sample flow through the cartridge was maintained at 5 mL min^{-1}. Once the sample passed through the cartridge, the cartridge was then dried for 10 mins by gently pulling air through it to remove any remaining water. The PPCPs were then eluted off the cartridge with 3 mL HPLC-grade methanol. The eluted sample was then completely dried under N_2 gas on a vacuum evaporation system (RapidVap, Labconco Kansas City, MO), reconstituted with LC-grade acetonitrile-water solution (1:1, v:v) to 1 mL, filtered through a 0.2 µm polytetrafluoroethylene (Thermo Scientific, Waltham, MA) syringe filter into a 2 mL glass amber vial (Agilent, Santa Clara, CA), and then screened for 138 PPCPs on a ultra-performance liquid chromatography-tandem mass spectrometry (UPLC/MS/MS) using method described below.

UPLC/MS/MS suspect screening for PPCPs

An ultra-performance liquid chromatography (UPLC) (1290, Agilent Technology, Santa Clara, CA) coupled with a tandem mass spectrometry (6490 triple quadrupole mass spectrometer, Agilent Technology, Santa Clara, CA) was used for the multi-compounds screening of 138 PPCPs. The PPCP suspect screening method was developed based on a compounds database and chromatographic method that was custom-developed by the Agilent Technology upon analysis of 140 analytical standards of compounds which were reported to be the most detected in environmental water. This database was developed using the same model of analytical column (Agilent Zorbax Extend C18 analytical column, 5 µm × 4.6 mm × 50 mm) and UPLC/MS/MS (Agilent 1290-6490) as that used in our study. This database contains specific compound identification criteria (i.e., retention time, precursor ions, product ions, collision energies) for the identification of each of the 138 PPCPs.

For the chromatographic section of the method, compounds were separated on the Agilent Zorbax Eclipse Plus C18 analytical column (2.1x100 mm, 1.8 µm). The temperature of the analytical column oven was kept at 40°C. Suspect PPCPs were screened using electrospray ionization (ESI) positive mode and then again negative mode on the mass spectrometer. For the ESI positive mode method, the analytical mobile phase A consisted of 5 mM Ammonium acetate + 0.02% acetic acid in water and analytical mobile phase B consisted of 95% acetonitrile in water. The mobile phase flow rate was 0.3mL min^{-1} under a gradient elution of 0 – 0.5 min, 5% B, 0.5 – 11 min, 5% B - 100% B, 11 - 13 min, 100%B, 13-13.1 min, 100B% - 5%B, and 13.1 – 16 min, 5%B. For the ESI negative mode method, the analytical mobile phase A consisted of 0.005% acetic acid in water and analytical mobile phase B consisted of 95% acetonitrile in water. The mobile phase flow rate was 0.3mL min^{-1} under a gradient elution of 0 – 0.5 min, 5% B, 0.5 – 6 min, 5% B - 100% B, 6 - 8 min, 100%B, 8-8.1 min, 100B% - 5%B, and 8.1 – 11 min, 5%B. The total sample injection volume for both methods was 20 µL.

For the mass spectrometry section of the method, electrospray ionization (ESI) and multiple reaction monitoring (MRM) mode was used. Each sample was analyzed separately in both positive and negative modes to detect oppositely charged ions. A compound in a sample was identified based on matching the masses of its precursor ion, product ion I, and product ion II with those listed in the database and further confirmed based on matching chromatographic retention time and peak shape between product ion I and II. In addition, if the signal to noise ratio of the chromatographic peak of one of the product ions for a suspected compound was less than 3, the identity of this compound was rejected.

For quality assurance and quality control (QA/QC), one laboratory blank (ultrapure Milli-Q water) and one spiked recovery sample (ultrapure Milli-Q water spiked with known concentrations of seven analytical standards: Carbamazepine, Erythromycin, Sulfamethazine, Chlorotetracycline, Tylosin, Sulfamethoxazole, and Triclosan) were extracted, cleaned up, concentrated, and analyzed along with the water samples for every 20 samples to monitor possible cross contamination of the compounds screened for and recovery of the spiked compounds. In addition, an instrument blank sample (UPLC mobile phase) was injected for every 20 samples to monitor possible instrument cross contamination. All analytes screened for were not detectable in any of the instrumental blanks or laboratory blanks, indicating free of cross contamination of those compounds during sample processing and analysis.

References

1. Gilchrist, C. L. M. & Chooi, Y.-H. clinker & clustermap.js: automatic generation of gene cluster comparison figures. *Bioinformatics* **37**, 2473–2475 (2021).
2. Shen, W., Le, S., Li, Y. & Hu, F. SeqKit: A Cross-Platform and Ultrafast Toolkit for FASTA/Q File Manipulation. *PLoS One* **11**, e0163962 (2016).
