# Supplementary material for: Selection and horizontal gene transfer underlie microdiversity-level heterogeneity in resistance gene fate during wastewater treatment
Source: Nat Commun. 2024 Jun 26;15:5412. doi: 10.1038/s41467-024-49742-8 (PMC11208604; doi:10.1038/s41467-024-49742-8)
Supplement: Supplementary file 3 — Description of Additional Supplementary Files [file 41467_2024_49742_MOESM3_ESM.pdf]

Description of Additional Supplementary Files

Supplementary Data 1: Study metadata, including sample names, number of sequencing reads, and groupings used throughout the study.

Supplementary Data 2: Metagenome assembled genome metadata including bin ID, checkm results, and gtdb-tk lineage predictions.

Supplementary Data 3: Supplementary data file containing counts of specific resistance gene fates with taxonomic metadata.

Supplementary Data 4: Complete suspect screening results for 140 pharmaceuticals and personal care products.

Supplementary Data 5: Predicted cross-phylum horizontal gene transfers.

Supplementary Data 6: Predicted cross-class horizontal gene transfers.

Supplementary Data 7: Predicted cross-order horizontal gene transfers.

Supplementary Data 8: Predicted cross-family horizontal gene transfers.

Supplementary Data 9: Predicted cross-genus horizontal gene transfers.

Supplementary Data 10: Example nanopore read alignments associated with the *mphA* encoding myxophage.

Supplementary Data 11: Bins classified to the phylum *Myxococcota*.

Supplementary Data 12: Functional annotation of bin204, gs. *Archangium* sp.
