# Supplementary material for: Selection and horizontal gene transfer underlie microdiversity-level heterogeneity in resistance gene fate during wastewater treatment
Source: Nat Commun. 2024 Jun 26;15:5412. doi: 10.1038/s41467-024-49742-8 (PMC11208604; doi:10.1038/s41467-024-49742-8)
Supplement: Supplementary file 13 — Supplementary Data 10 [file 41467_2024_49742_MOESM13_ESM.docx]

**Supplementary Data 10 – Nanopore read alignment to phage genome bearing *mphA***

RID: JXTTA6E6114

Job Title:Nucleotide Sequence

Program: BLASTN

Query: None ID: lcl|Query_50208(dna) Length: 1691

Subject #1:CP026228.1:580637-581580 Aeromonas sp. ASNIH1 chromosome, complete genome ID: lcl|Query_50210 Length: 944

Subject #2:CP064980.1:16820-17479 Myxococcales bacterium isolate Fred_18-Q3-R57-64_BAT3C.417 chromosome ID: lcl|Query_50211 Length: 660

Sequences producing significant alignments:

Scientific Common Max Total Query E Per. Acc.

Description Name Name Taxid Score Score cover Value Ident Len Accession

CP026228.1:580637-581580 Aeromonas sp. ASNIH1 chromosome,... 0 859 859 54% 0.0 83.85 944 Query_50210

CP064980.1:16820-17479 Myxococcales bacterium isolate... 0 652 652 39% 0.0 84.56 660 Query_50211

Alignments:

>CP026228.1:580637-581580 Aeromonas sp. ASNIH1 chromosome, complete genome

Sequence ID: Query_50210 Length: 944

Range 1: 1 to 944

Score:859 bits(465), Expect:0.0,

Identities:810/966(84%), Gaps:66/966(6%), Strand: Plus/Minus

Query 714 GCCGCTGCCGCCCATCGTGCCGAACCACTGGGAGCTGGGCCTCAGCGA-C-CTGG-GAAA 770

||| ||||||||| | | ||||||||||||||||||| || || ||| | | || | |

Sbjct 944 GCC-CTGCCGCCCGTTGCTCCGAACCACTGGGAGCTGGACCCCAACGATCTCGGGTGTGA 886

Query 771 CCACGGACCTGCATTGAGGGGGC--CGGG-CGT----CG--T--CAGGAGTGGTTGATTA 819

| | ||||||| | ||||||||| ||| ||| || | || ||| | |||| ||

Sbjct 885 CGATGGACCTGTACTGAGGGGGCTGTGGGTCGTACCCCGATTCGCATGAG-GATTGAATA 827

Query 820 TAACCGACGCGACTGTTATAAATT-G-CGCCAGATCCGTCGA-TCCTCTGGAGCGATTCA 876

||||||||| |||||||||| | | || | | ||||| | |||| |||| || |||

Sbjct 826 TAACCGACGTGACTGTTATATTTAGGTGGCTAAACCCGTCAAGCCCTCAGGAGTGAATCA 767

Query 877 TGACCGCAG-C-CGTAACCGTCGATACCTCCCAACTGCACGCGCT--C-GCCGGGCATGG 931

|||||| || | || |||| |||||||||||||||| |||| || | ||| | ||| |

Sbjct 766 TGACCGTAGTCACG--ACCGCCGATACCTCCCAACTGTACGCACTTGCAGCCCGACAT-G 710

Query 932 GGCT--GGTTTCATGGCCCACTGACCGTGAATGAGCTTGGGCTCGACTATCGG-TCGTGA 988

|||| | | |||||||| ||||| || ||||||||||||||||||||| || ||||||

Sbjct 709 GGCTCAAGCTCCATGGCCCGCTGACTGTCAATGAGCTTGGGCTCGACTATAGGATCGTGA 650

Query 989 TTGCCGCCGTCGACGATGGACGTCGGTGGGTGCTGCGCATCCCGCGCCG-GCCGAGAGTG 1047

| ||| ||||||||||||||||||||||||||||||||||||||||||| |||||| ||

Sbjct 649 TCGCCACCGTCGACGATGGACGTCGGTGGGTGCTGCGCATCCCGCGCCGAGCCGAG-GTA 591

Query 1048 AGCGCGAAGGTCGAGCCAGAGGCGCGGGTGCTGGCGATGCTCAA--AGCGCTTGCCGTTC 1105

|||||||||||||| |||||||||||||||||||| |||||||| | ||| ||||||||

Sbjct 590 AGCGCGAAGGTCGAACCAGAGGCGCGGGTGCTGGCAATGCTCAAGAATCGCCTGCCGTTC 531

Query 1106 GCGGTGCCGGACTGGCGCGTGGCCAACGCCGAGCTCGTTGCCTATCCCATGCTCGAGGAC 1165

|||||||||||||||||||||||||||||||||||||||||||||||||||||||| |||

Sbjct 530 GCGGTGCCGGACTGGCGCGTGGCCAACGCCGAGCTCGTTGCCTATCCCATGCTCGAAGAC 471

Query 1166 TCGACTGCGATCGTCCTCCAACCTGGCTCGTC-GCG-CCGACTGGGTCGTGCCGCCGGGC 1223

||||||||||| ||| |||| ||||| ||||| || |||||||||||||||||| || |

Sbjct 470 TCGACTGCGATGGTCATCCAGCCTGGTTCGTCCACGCCCGACTGGGTCGTGCCGCAGGAC 411

Query 1224 T-GGA---CTTCGCGGAGAGCTTCGCGACGGCGCTCGCCGCTC-GC--GCCGT-TCCGTC 1275

| ||| ||||||||||||||||||||| ||||||||||| | || ||||| || |

Sbjct 410 TCGGAGGTCTTCGCGGAGAGCTTCGCGACCGCGCTCGCCGCCCTGCATGCCGTCCCCATT 351

Query 1276 TCCGCCGCCGTAGATGCGGGGATGCTCATCCGCACGCCGCCGCAGGCCCGTC-GGAGGT- 1333

||||||||||| |||||||||||||||||||| || ||| |||||||||||| | ||||

Sbjct 350 TCCGCCGCCGTGGATGCGGGGATGCTCATCCGTACACCGACGCAGGCCCGTCAGAAGGTG 291

Query 1334 CACGACGACGTTGAGCGCGTCCGACGCGAGTTCGTGGTGAACGACAAGCGCCTCCACCGA 1393

|||||||||||| ||||||||||||||||||||||||||||||||||||||||||||

Sbjct 290 GCCGACGACGTTGACCGCGTCCGACGCGAGTTCGTGGTGAACGACAAGCGCCTCCACCGG 231

Query 1394 TGGCAGCGCTGGCTCGACGACGATTCGTCGTCGGCCGGATTTCTGCTGCGTGGTGGCGTA 1453

||||||||||||||||||||||||||||||| |||| ||||||| | |||||| | ||

Sbjct 230 TGGCAGCGCTGGCTCGACGACGATTCGTCGT-GGCCAGATTTCT-C--CGTGGT-G-GTG 177

Query 1454 CGTCGGCGATCTCTACGTGGGCCATGTGCTCGTCGACAACACGGAGCGCGTCAGCGGGAT 1513

| | ||||||||||||||||||||||||||| ||||||||||||||||||||||||||||

Sbjct 176 CAT-GGCGATCTCTACGTGGGCCATGTGCTCATCGACAACACGGAGCGCGTCAGCGGGAT 118

Query 1514 GATCGACTGGAGCGAGGCCCCGCGAGTTGGACGATGACCTCCCCATCGACATGGCCTCGC 1573

|||||||||||||||||||| ||| ||| | ||||| |||||||||||||| |||

Sbjct 117 GATCGACTGGAGCGAGGCCC-GCG--TTG-A---TGACCCTGCCATCGACATGGCCGCGC 65

Query 1574 ACCTCATGGTCTTTGGCGAGGCGGGGCTC---A-CTTCCTCCTCACGTACGAGAAGCGGC 1629

|||| ||||||||||| || | ||||||| | ||||||||||||| || | |||||

Sbjct 64 ACCTTATGGTCTTTGGTGAAGAGGGGCTCGCGAAGCTCCTCCTCACGTATGA-A-GCGGC 7

Query 1630 TGGAGG 1635

|| ||

Sbjct 6 CGGTGG 1

>CP064980.1:16820-17479 Myxococcales bacterium isolate Fred_18-Q3-R57-64_BAT3C.417 chromosome

Sequence ID: Query_50211 Length: 660

Range 1: 1 to 660

Score:652 bits(353), Expect:0.0,

Identities:575/680(85%), Gaps:24/680(3%), Strand: Plus/Minus

Query 73 TCGGCGTGACGGACCTCTTCGGCAACGTCGTCGTCGGCCCCTACGACCGCGCGAGCTCCG 132

||||||| |||||||| ||||||||||||||| ||| || ||||||||||||| || |

Sbjct 660 TCGGCGTCACGGACCTGTTCGGCAACGTCGTCCTCGCGCCTTACGACCGCGCGACCTTCA 601

Query 133 CCCGGTTCCCGGCCCGCGCG-CCGC-GACCCGCCGCTTCGACCTGTGGCGCATGCTGCCG 190

| |||||| | |||||||| |||| ||| |||||||||||||| ||||| ||||| ||

Sbjct 600 CGGGGTTCC-GCCCCGCGCGACCGCAGACGCGCCGCTTCGACCTCTGGCGGATGCTCCCC 542

Query 191 AAGCACAACCGACGAAGCGA-CCGACCGGAGACCTGTTCCGGTTCATCGCGTGTCTGCAG 249

||||||||||| || ||| | |||||| ||||| |||||||||||||| || ||||||

Sbjct 541 AAGCACAACCGCCGCGACGATCAGACCGGGGACCTTTTCCGGTTCATCGCCTGCCTGCAG 482

Query 250 GAGGTGACGGACCTCCTGCTCGCCGACGTGGACCGCTGGCCCGACGTCTTCTTCGATCTG 309

|||||||| ||||| ||||| ||||||||||| |||||||| | | | ||||||| ||

Sbjct 481 GAGGTGACCGACCTGCTGCTGGCCGACGTGGATCGCTGGCC-G--GACATCTTCGA-CT- 427

Query 310 GGGAGCGCGCGCGCCCGAGGCCTTCATCGATCTCATCCTGCGCGATCTCGGCAACCCATT 369

|| ||||||||||||||||||| |||| |||||||| ||||| ||||||||||| ||

Sbjct 426 TCGA--GCGCGCGCCCGAGGCCTTCCTCGACCTCATCCTCCGCGACCTCGGCAACCCGTT 369

Query 370 CCCGTTCGAGCTCGACGTGCTGGGCAAGCGTCGTCTTGCGTCGGTGCTCGTCGAGATGTA 429

|||||||||||||||| |||| ||||| || || |||||||||||||||||||||||

Sbjct 368 CCCGTTCGAGCTCGACACCATGGGGAAGCGCCGACTCGCGTCGGTGCTCGTCGAGATGTA 309

Query 430 CCGACAGAAGGGCACGGCCAAGGGTATCCAGAACGCGATCCGCTTCTTCCTCGGCATCGA 489

||| |||||||| ||||| ||||| |||||||||||||||||||||||||||||||||||

Sbjct 308 CCGGCAGAAGGGGACGGCGAAGGGCATCCAGAACGCGATCCGCTTCTTCCTCGGCATCGA 249

Query 490 CATCTCCGCCATCACGCCGTTCGAACTCACGCCAACGTTTTACGCTCGCCCTCGGCGGGT 549

|||||| |||||| |||||||| ||| | || | | |||||| |||||||| ||

Sbjct 248 CATCTCGGCCATCTCGCCGTTC-AACGC-CG--A-C-----ACGCTCATCCTCGGCGAGT 199

Query 550 CCGAGCTGGGCGTCGACTGGGTGCTCGGCCCCTCCGACCGCTTCGCGCGCTACGCCTTCA 609

||||||||||| |||||||||| ||||| ||||| ||||||||||| || ||||||||||

Sbjct 198 CCGAGCTGGGCATCGACTGGGTCCTCGGGCCCTCGGACCGCTTCGCCCGGTACGCCTTCA 139

Query 610 ACGTCGTGGTCGCGCGTATCCTCAGCGGACCGGAAGCGTCGCCAGCTCCGGGCCATCGTC 669

|||||| ||||||||| ||||| || || || |||| || |||||||| |||||||||

Sbjct 138 ACGTCGAGGTCGCGCGCATCCTG-GCCGATCGCGAGCGCCGGCAGCTCCGCGCCATCGTC 80

Query 670 G-GTGCCCTGAAGCCCGCGCACACGCACTTCGTGGACCTCGTCGAGCCGCTGCCGCCCAT 728

| || ||| |||||||||||||| |||||||| || || ||||||||||| ||||||||

Sbjct 79 GAGTA-CCTCAAGCCCGCGCACACCCACTTCGTCGATCTGGTCGAGCCGCTCCCGCCCAT 21

Query 729 CGTGCCGAACCACTGGGAGC 748

| ||||||||||||||||||

Sbjct 20 CCTGCCGAACCACTGGGAGC 1

**Raw fastq read:**

@6de60e4c-c959-472c-93f4-1fe535ef4dd0 runid=f4c844d6bbe51d691623b437e8130a7833033316 sampleid=2020_09_05_ORANGE_327-410 read=64680 ch=253 start_time=2020-09-07T13:46:15Z barcode=barcode06

CGATGCTCCTTCGTTCAGTCTATCCTGTTGCTAAGGTTGAGACTACTTCTGCCTTGCGAGAACAGCACCTCATCGGCGTGACGGACCTCTTCGGCAACGTCGTCGTCGGCCCCTACGACCGCGCGAGCTCCGCCCGGTTCCCGGCCCGCGCGCCGCGACCCGCCGCTTCGACCTGTGGCGCATGCTGCCGAAGCACAACCGACGAAGCGACCGACCGGAGACCTGTTCC

GGTTCATCGCGTGTCTGCAGGAGGTGACGGACCTCCTGCTCGCCGACGTGGACCGCTGGCCCGACGTCTTCTTCGATCTGGGGAGCGCGCGCGCCCGAGGCCTTCATCGATCTCATCCTGCGCGATCTCGGCAACCCATTCCCGTTCGAGCTCGACGTGCTGGGCAAGCGTCGTCTTGCGTCGGTGCTCGTCGAGATGTACCGACAGAAGGGCACGGCCAAGGGTATCC

AGAACGCGATCCGCTTCTTCCTCGGCATCGACATCTCCGCCATCACGCCGTTCGAACTCACGCCAACGTTTTACGCTCGCCCTCGGCGGGTCCGAGCTGGGCGTCGACTGGGTGCTCGGCCCCTCCGACCGCTTCGCGCGCTACGCCTTCAACGTCGTGGTCGCGCGTATCCTCAGCGGACCGGAAGCGTCGCCAGCTCCGGGCCATCGTCGGTGCCCTGAAGCCCGCG

CACACGCACTTCGTGGACCTCGTCGAGCCGCTGCCGCCCATCGTGCCGAACCACTGGGAGCTGGGCCTCAGCGACCTGGGAAACCACGGACCTGCATTGAGGGGGCCGGGCGTCGTCAGGAGTGGTTGATTATAACCGACGCGACTGTTATAAATTGCGCCAGATCCGTCGATCCTCTGGAGCGATTCATGACCGCAGCCGTAACCGTCGATACCTCCCAACTGCACGC

GCTCGCCGGGCATGGGGCTGGTTTCATGGCCCACTGACCGTGAATGAGCTTGGGCTCGACTATCGGTCGTGATTGCCGCCGTCGACGATGGACGTCGGTGGGTGCTGCGCATCCCGCGCCGGCCGAGAGTGAGCGCGAAGGTCGAGCCAGAGGCGCGGGTGCTGGCGATGCTCAAAGCGCTTGCCGTTCGCGGTGCCGGACTGGCGCGTGGCCAACGCCGAGCTCGTTG

CCTATCCCATGCTCGAGGACTCGACTGCGATCGTCCTCCAACCTGGCTCGTCGCGCCGACTGGGTCGTGCCGCCGGGCTGGACTTCGCGGAGAGCTTCGCGACGGCGCTCGCCGCTCGCGCCGTTCCGTCTCCGCCGCCGTAGATGCGGGGATGCTCATCCGCACGCCGCCGCAGGCCCGTCGGAGGTCACGACGACGTTGAGCGCGTCCGACGCGAGTTCGTGGTGAA

CGACAAGCGCCTCCACCGATGGCAGCGCTGGCTCGACGACGATTCGTCGTCGGCCGGATTTCTGCTGCGTGGTGGCGTACGTCGGCGATCTCTACGTGGGCCATGTGCTCGTCGACAACACGGAGCGCGTCAGCGGGATGATCGACTGGAGCGAGGCCCCGCGAGTTGGACGATGACCTCCCCATCGACATGGCCTCGCACCTCATGGTCTTTGGCGAGGCGGGGCTCA

CTTCCTCCTCACGTACGAGAAGCGGCTGGAGGGGTGCTGTTCTGGCGAAGGCGAAGGTCGTCTTAACCTTAGCGATCGTCGCCGACTG

+

%(/)%',(.'4$388&#+0$$$$$&*((,85>??=@B?31140;)7899;)2,9*%$#$&-25+85/<=C@A%%?CBDADD5=>E?@E>;;*5>,+-/-..,72:;?AD?;74;88;@<>608;--40;-55&24$)0($-9:;2277?<5+0++//1-4=FDBDCHKGGE>?>G@JAA@CC>E<<EEB>A69?3;;;B>E=942+))1')*7861&,)*)%68:5F?B

BGDGBCB<D<B>>?A@DCE;;IC=?>B;E@<9=<<<?77B:%*65B<>?8>@>A94588$>:960%&4-9.()+3<;55(+,4557%-/,2(,*.97A=GAA>@@)&&/**(55.-/:87=@@AA>;??AA=??=<C;JF>?<?HHA<C><EC4GE:@CDCCGG>?@EE8E9CA?==:8DC@=E>D>C@DB<DADB?G7BCBH;CJ>EDA>IFCBBEF7/44?DEDC9E

>FGKD@@@KH@BNDC=FGOF<E@<?B?B>HFC=8;5@>?>IGCEFAAB<@A@2**1)%&%($+'%-*%%-*#(,71>8):E8@5)(&$#%%*.,1/+:1EH@++@<A@>*>=;<7<<GB===;@@6;>5;GBEKAGCA=95@A=HADEFH?LIAGEC==EED@@?=AA554/*&#30313:;*8+6-6--=/@I=C?F>@<CG=C<:824&%)%$&;:<?1B30C=CB?

?@BBCGDIGGJ=B@78F:==:EF@DF@?>D?DD=;@D9@//4:ACH<;FD<>;HG6?;@?>A)B?3;>596//><A:<37#3#%2%&=;:<@:$,*8?/*878=9+,==E>8E8>AD@BB@:?>7<10:0,&44=B;DA;6@C?<=BEIA>:885528:&(&(7226@B:@EGB<>@DHCC?>EHPHFGBE?>@A>GJH=FEHHE<GFBFA=978>4>A=CADEJI;@>

G?D=1/72:88763.7645)%$23499:598;8<ADG?<BEFFDBLHL=>?;>CAA=)<93(&&E>46@@=;=@251%/4=@<<F?=ID?@E@9>>B>=8=@:>>>@?ECBC=@?CB)3(-614$$%#$&%23?<<<II@DD<==53903;;DEB<=DJAB@<F?99962$$%235./;<>C>A?BGGEI>H?>AA:A;<<A=>FFABB;;CC<EABG?=>=<;5)G78

76<524?/+?B?F>9<=6-)+$<9:<:=BCA@@C:7E>BBC>A?D;==5:;>0:2=9->>?$??<203228(%31:998*58,62==C?DI;DCCEG:9)554:=<=9</4*()##%&6527,&0*/,7665?7..,:757-5;0.:99+444223899>6B?AC:A@C878C;BB2<6=<*&.&%+,(-27:8459=A3597G3=;>::?@=?>CGDCE@?<9AAD@>

9<A=CB0)@D==8@GIDGFCCB@@BD94>78@:6/:7)),9>>@98:757(21)/$)&#,'&,/#'$$''&((&%%-,$'%%.@F7=:9:3;;0;432BG:BDCBDC@C;D?DDCB>>G:A=<97C9:**;5EBAF@B@>E?8?:9>47?;500/6%,4/0&#&'(3$/-*'+--4550'&79;=?1E?3?A@D>@9BFB@@@,(),<855?BGA<=C>;86B?533'%

$%42:;59=AA@@@?7''3CD@@?ECDHBD><:9?<46-*.$"$%$$385+$(&=,56$0@;7:1-(1)4$&%%,,$%%$%*$##$)"
