# Supplementary material for: Selection and horizontal gene transfer underlie microdiversity-level heterogeneity in resistance gene fate during wastewater treatment
Source: Nat Commun. 2024 Jun 26;15:5412. doi: 10.1038/s41467-024-49742-8 (PMC11208604; doi:10.1038/s41467-024-49742-8)
Supplement: Supplementary file 16 — Reporting Summary [file 41467_2024_49742_MOESM16_ESM.pdf]

nature portfolio

Corresponding author(s): Amy Pruden and Peter Vikesland
Last updated by author(s): Jun 4, 2024

Reporting Summary

Nature Portfolio wishes to improve the reproducibility of the work that we publish. This form provides structure for consistency and transparency in reporting. For further information on Nature Portfolio policies, see our Editorial Policies and the Editorial Policy Checklist.

Statistics

For all statistical analyses, confirm that the following items are present in the figure legend, table legend, main text, or Methods section.

n/a	Confirmed	
☐	☒	The exact sample size (*n*) for each experimental group/condition, given as a discrete number and unit of measurement
☐	☒	A statement on whether measurements were taken from distinct samples or whether the same sample was measured repeatedly
☐	☒	The statistical test(s) used AND whether they are one- or two-sided<br>*Only common tests should be described solely by name; describe more complex techniques in the Methods section.*
☒	☐	A description of all covariates tested
☐	☒	A description of any assumptions or corrections, such as tests of normality and adjustment for multiple comparisons
☐	☒	A full description of the statistical parameters including central tendency (e.g. means) or other basic estimates (e.g. regression coefficient) AND variation (e.g. standard deviation) or associated estimates of uncertainty (e.g. confidence intervals)
☐	☒	For null hypothesis testing, the test statistic (e.g. *F*, *t*, *r*) with confidence intervals, effect sizes, degrees of freedom and *P* value noted<br>*Give P values as exact values whenever suitable.*
☒	☐	For Bayesian analysis, information on the choice of priors and Markov chain Monte Carlo settings
☒	☐	For hierarchical and complex designs, identification of the appropriate level for tests and full reporting of outcomes
☒	☐	Estimates of effect sizes (e.g. Cohen's *d*, Pearson's *r*), indicating how they were calculated

Our web collection on statistics for biologists contains articles on many of the points above.

Software and code

Policy information about availability of computer code

Data collection: No software was used for data collection.

Data analysis:
bbmap version 35.85
diamond v. 0.9.14
bowtie2 v. 2.2.5
OPERA-MS v0.8.3
hybridSPAdes v. 3.15.0
MaxBin v. 2.2.7
MetaBat2 v. 2:2.15
drep v. v3.2.2
minimap2 v. 2.24-r1122
prodigal v. 2.6.3
gtdb-tk V. 2.2.6
Kairos derep-detect v. 0.1
Kairos assess v. 0.1
guppy v.3.2.10

custom scripts associated with data analysis are available here:
https://github.com/clb21565/metagenomics/tree/main/HospitalEffluentProject

and here:
https://github.com/clb21565/kairos

For manuscripts utilizing custom algorithms or software that are central to the research but not yet described in published literature, software must be made available to editors and reviewers. We strongly encourage code deposition in a community repository (e.g. GitHub). See the Nature Portfolio guidelines for submitting code & software for further information.

Data

Policy information about availability of data

All manuscripts must include a data availability statement. This statement should provide the following information, where applicable:

- Accession codes, unique identifiers, or web links for publicly available datasets
- A description of any restrictions on data availability
- For clinical datasets or third party data, please ensure that the statement adheres to our policy

All sequencing reads have been deposited to the SRA under BioProject PRJNA1020581. MAGs can be accessed at https://zenodo.org/records/10028566.

Research involving human participants, their data, or biological material

Policy information about studies with human participants or human data. See also policy information about sex, gender (identity/presentation), and sexual orientation and race, ethnicity and racism.

Reporting on sex and gender	N/A
Reporting on race, ethnicity, or other socially relevant groupings	N/A
Population characteristics	N/A
Recruitment	N/A
Ethics oversight	N/A

Note that full information on the approval of the study protocol must also be provided in the manuscript.

Field-specific reporting

Please select the one below that is the best fit for your research. If you are not sure, read the appropriate sections before making your selection.

☐ Life sciences ☐ Behavioural & social sciences ☒ Ecological, evolutionary & environmental sciences

For a reference copy of the document with all sections, see nature.com/documents/nr-reporting-summary-flat.pdf

Ecological, evolutionary & environmental sciences study design

All studies must disclose on these points even when the disclosure is negative.

Study description

The current study used lab-scale sequencing batch reactors to investigate microbial ecology, horizontal gene transfer, and antibiotic contamination in activated sludge. Six independent reactors were operated in parallel on a 12 hr cycle for a period of ~3 months until they showed stable removal of organic carbon. At this time, the influent feed to three of the reactors was augmented to contain 10% of hospital sewage to 90% municipal sewage. Three different sample types were taken from the reactors for metagenomics, chemical suspect screening and physical-chemical analysis. Samples were taken during aeration (associated samples called "activated sludge" or "mixed liquor" that included solids and liquid wastewater; samples of effluent (treated wastewater/reactor supernatant following settling) or influent (i.e., the feed wastewater). Influent (feed) wastewater was collected from a local wastewater treatment plant every 5 days for the length of the study. Reactors were sampled every 2-3 days and treated as biological replicates for the 0% or 10% hospital sewage conditions.

For comparisons across conditions, we controlled for the reactor (i.e., grouped data points across time points according to which replicate reactor the data corresponded to). Conditions were whether the influent to the reactor contained 0% or 10% hospital sewage; the phase of the experiment (before spiking, initial period following spiking, and after spiking).

Research sample

Wastewater samples from the reactors were taken during aeration (associated samples called "activated sludge" or "mixed liquor" that included solids and liquid wastewater; samples of effluent (treated wastewater/reactor supernatant following settling) or influent (i.e., the feed wastewater). Influent (feed) wastewater was collected from a local wastewater treatment plant every 5 days for the length of the study. Reactors were sampled every 2-3 days and treated as biological replicates for the 0% or 10% hospital sewage conditions.

Sampling strategy

Sampling efforts were chiefly constrained by the operating volume of the reactors (3 L) and the manual labor involved in their

Sampling strategy	maintenance. As each reactor represented a distinct, independent microbiome, we deemed our sampling strategy to be sufficient for addressing the impact of hospital sewage on activated sludge performance under realistic conditions.
Data collection	Wastewater samples were taken and analyzed for physical chemical and operational parameters as listed below. The HACH reactor digestion method (method 8000, HACH, Loveland, CO) was used for COD and sCOD. Samples for sCOD analysis were filtered through 0.45-µm filter prior to digestion. Ammonia was tested using the HACH salicylate method (Method 8155 HACH, Loveland, CO). Alkalinity, TSS, volatile suspended solids (VSS), and SVI were tested according to Standard Methods for the Examination of Water and Wastewater (methods 2320, 2540, and 213 respectively).<br>Sampling for DNA was performed by filtering 20-100 mL of wastewater onto 0.22 um mixed ester cellulose filters and exact volumes calculated by weighing the sample before and after filtration and calculating the difference.<br>Associated measurements were recorded in lab notebooks.<br>Data were collected by CLB and AMM.
Timing and spatial scale	Metagenomic data that were the primary subject of this manuscript were collected from 03/20/2020 - 04/10/2020. Sampling was performed for metagenomics 9 times. The time between each sampling event was: 2 days, 2 days, 3 days, 2 days, 2 days, 3 days, 4 days, and 10 days. Sample collection dates were chosen based on the solids retention time of 5 days and as feasible. Samples were collected from reactors with operating volumes of 3 liters.
Data exclusions	No data were excluded from analyses.
Reproducibility	Due to time, labor, and other constraints, no attempts at replication were made beyond the initial replicate reactor study design. Replicate reactors run in parallel remained similar in terms of operational performance metrics.
Randomization	The study involved repeated sampling of biological replicate reactors and thus did not require randomization. In our analyses, we controlled for factors such as reactor identity across time points to control for baseline variability between reactors.
Blinding	The study involved repeated sampling of biological replicate reactors for metagenomic sequencing. As such, blinding of data would not be relevant.

Did the study involve field work? ☐ Yes ☒ No

Reporting for specific materials, systems and methods

We require information from authors about some types of materials, experimental systems and methods used in many studies. Here, indicate whether each material, system or method listed is relevant to your study. If you are not sure if a list item applies to your research, read the appropriate section before selecting a response.

Materials & experimental systems

n/a	Involved in the study
☒	☐ Antibodies
☒	☐ Eukaryotic cell lines
☒	☐ Palaeontology and archaeology
☒	☐ Animals and other organisms
☒	☐ Clinical data
☒	☐ Dual use research of concern
☒	☐ Plants

Methods

n/a	Involved in the study
☒	☐ ChIP-seq
☒	☐ Flow cytometry
☒	☐ MRI-based neuroimaging

Plants

Seed stocks	*Report on the source of all seed stocks or other plant material used. If applicable, state the seed stock centre and catalogue number. If plant specimens were collected from the field, describe the collection location, date and sampling procedures.*
Novel plant genotypes	*Describe the methods by which all novel plant genotypes were produced. This includes those generated by transgenic approaches, gene editing, chemical/radiation-based mutagenesis and hybridization. For transgenic lines, describe the transformation method, the number of independent lines analyzed and the generation upon which experiments were performed. For gene-edited lines, describe the editor used, the endogenous sequence targeted for editing, the targeting guide RNA sequence (if applicable) and how the editor was applied.*
Authentication	*Describe any authentication procedures for each seed stock used or novel genotype generated. Describe any experiments used to assess the effect of a mutation and, where applicable, how potential secondary effects (e.g. second site T-DNA insertions, mosiacism, off-target gene editing) were examined.*
